# Clinical features of Hispanic thyroid cancer cases and the role of known genetic variants on disease risk

**DOI:** 10.1097/MD.0000000000004148

**Published:** 2016-08-12

**Authors:** Ana P. Estrada-Florez, Mabel E. Bohórquez, Ruta Sahasrabudhe, Rodrigo Prieto, Paul Lott, Carlos S. Duque, Jorge Donado, Gilbert Mateus, Fernando Bolaños, Alejandro Vélez, Magdalena Echeverry, Luis G. Carvajal-Carmona

**Affiliations:** a Grupo de Citogenética, Filogenia y Evolución de Poblaciones, Facultad de Ciencias y Facultad de Ciencias de la Salud, Universidad del Tolima, Ibagué, Colombia; b Genome Center and Department of Biochemistry and Molecular Medicine, School of Medicine, University of California, Davis, CA; c Hospital Pablo Tobón Uribe, Medellín; d Hospital Federico Lleras Acosta, Ibagué; e Hospital Hernando Moncaleano Perdomo, Neiva; f Fundación de Genética y Genómica, Medellín, Colombia.

**Keywords:** association, Hispanics, risk variants, thyroid cancer

## Abstract

Thyroid cancer (TC) is the second most common cancer among Hispanic women. Recent genome-wide association (GWA) and candidate studies identified 6 single nucleotide polymorphisms (SNPs; rs966423, rs2439302, rs965513, rs6983267, rs944289, and rs116909374), associated with increased TC risk in Europeans but their effects on disease risk have not been comprehensively tested in Hispanics. In this study, we aimed to describe the main clinicopathological manifestations and to evaluate the effects of known SNPs on TC risk and on clinicopathological manifestations in a Hispanic population.

We analyzed 281 nonmedullary TC cases and 1146 cancer-free controls recruited in a multicenter population-based study in Colombia. SNPs were genotyped by Kompetitive allele specific polymerase chain reaction (KASP) technique. Association between genetic variants and TC risk was assessed by computing odds ratios (OR) and confidence intervals (CIs).

Consistent with published data in U.S. Hispanics, our cases had a high prevalence of large tumors (>2 cm, 43%) and a high female/male ratio (5:1). We detected significant associations between TC risk and rs965513A (OR = 1.41), rs944289T (OR = 1.26), rs116909374A (OR = 1.96), rs2439302G (OR = 1.19), and rs6983267G (OR = 1.18). Cases carried more risk alleles than controls (5.16 vs. 4.78, *P* = 4.8 × 10^−6^). Individuals with ≥6 risk alleles had >6-fold increased TC risk (OR = 6.33, *P* = 4.0 × 10^−6^) compared to individuals with ≤2 risk alleles. rs944289T and rs116909374A were strongly associated with follicular histology (ORs = 1.61 and 3.33, respectively); rs2439302G with large tumors (OR = 1.50); and rs965513A with regional disease (OR = 1.92).

To our knowledge, this is the first study of known TC risk variants in South American Hispanics and suggests that they increase TC susceptibility in this population and can identify patients at higher risk of severe disease.

## Abstract 2

Supplemental Digital Content is available in the text

## 1 Introduction

Thyroid cancer (TC) is the most common endocrine malignancy, with about 300,000 new cases diagnosed annually across the world and worldwide it is the eighth most common cancer in women. ^[1]^ The American Cancer Society (ACS) estimates that during 2016, in the United States, there will be 62,450 new TC cases (47,230 in women and 15,220 in men) and ∼1950 TC-related deaths. ^[2]^ Based on the latest ACS report in U.S. Hispanic women, TC is now the second most commonly diagnosed malignancy with an age standardized rate (ASR) of 17.2 cases per 100,000 population and representing 9% of all incident female cancer cases in this U.S. minority. ^[3]^ Only breast cancer, representing 29% (estimated at 19,800 new female cases per year) of all newly diagnosed female cases, is more commonly diagnosed than TC in U.S. Hispanic women. ^[3]^ In U.S. Hispanic women, the estimated number of new TC cases (6000 per year) is now higher than that of colorectal cancer (5300 new cases), uterine cancer (5100 new cases per year) and cancer of the lung and bronchus (4600 new cases per year. For details, see fig. 1 in Ref. ^[3]^). In Colombia, a country with a predominant Hispanic population,[ ^4^ ^5^] the TC ASRs range from 1.2 in men and 8.6 in women, representing the fifth most commonly diagnosed cancer among women from this South American country. ^[1]^

Epidemiological studies have identified several potentially important TC risk factors such as female gender, a personal history of thyroid adenomas, dietary iodine imbalance, and exposure to radiation. ^[6]^ Interestingly, several studies have also suggested that a family history of TC is another major risk factor for this malignancy. For example, a study in the Utah Population Database showed that relatives of TC patients were 8 times more likely to have the disease themselves when compared to the general population. ^[7]^ In another study, carried out in the Swedish Family-Cancer Database, the reported standardized incidence ratio (SIR) in TC was ∼7, higher than for any of the cancer types investigated in the study. ^[8]^ These studies therefore suggest that genetic studies are likely to be very important in estimating the risk of developing TC.

Despite the strong familial risk in TC, the genetics of this malignancy remains relatively unexplored. Few studies have suggested that common low penetrance variants account for a significant fraction of TC risk. ^[9]^ Recent genome-wide association (GWA) and candidate studies in Europeans have identified TC risk alleles on chromosomes 2q35 (rs966423), 8p12 (rs2439302), 8q24 (rs6983267), 9q22 (rs965513), and 14q13 (rs944289 and rs116909374). [^10^ ^11^ ^12^ ^13^] The effects of some of these known TC variants have been tested in independent populations and so far, most of these variants have been replicated in populations of British, ^[14]^ Japanese, ^[15]^ and Chinese[ ^16^ ^17^] ancestry. However, to our knowledge, these known single nucleotide polymorphisms (SNPs) have not been comprehensively tested in any South American population. Moreover, TC risk stratified by clinical variables has not been thoroughly investigated in Hispanics. Therefore, the goal of the present study was to examine the associations between TC risk and known TC variants and stratify it with clinicopathological features in a multicenter population-based Hispanic case–control study.

## 2 Materials and methods

### 2.1 Study population

A total of 281 histologically verified cases with nonmedullary TC (48 males and 233 females) were recruited, between 2010 and 2014, in a multicenter study in Colombia.[ ^13^ ^18^] For all analyses, we grouped the follicular variant of papillary TC with follicular TC (FTC) in accordance with their morphological ^[19]^ and molecular similarities. ^[20]^ Our study included 172 cases with papillary TC (PTC), 109 with FTC and 1146 population controls matched with cases by sex and geographical origin (235 males and 911 females). Population controls were cancer-free and did not have family history of cancer in first- and second-degree relatives at the time of recruitment. After providing informed consent, cases and controls were interviewed in-person by trained research nurses with a questionnaire that collected information on local ancestry, sociodemographics, disease presentation, personal and family history of cancer, lifestyle, and TC risk factors. The research protocol used in the study adhered to the Helsinki declaration and was approved by the Ethics committees from University of Tolima, Hospital Federico Lleras Acosta, Hospital Hernando Moncaleano, and Hospital Pablo Tobón Uribe.

### 2.2 SNPs genotyping

Genomic DNA was isolated from the whole blood samples using the Promega Maxwell16 system. We genotyped 6 SNPs (rs966423, rs2439302, rs6983267, rs965513, rs116909374, and rs944289) in these samples using the competitive allele-specific PCR with KASP genotyping system (LGC Genomics, London, England) with reaction conditions previously reported. ^[14]^ Genotyping call rates were >98% for all the markers (data not shown) and replicates with known genotypes were in full concordance across all the assays.

### 2.3 Statistical analysis

#### 2.3.1 Analyses of clinicopathological characteristics

For all 281 cases, we had information on sex (male, female), histological subtype (PTC, FTC), and family history of cancer in first- and second-degree relatives (present, absent). For 241 of these patients, we also had information about tumor size (small ≤2 cm, large >2 cm), focality (unifocal, multifocal), bilaterality (absent, present), capsule invasion (absent, present), vascular invasion (absent, present), extrathyroidal extension (absent, present), lymph node metastasis (absent, present), and distant metastasis (absent, present). We stratified these variables by age at diagnosis (diagnosed before age 45 years and at/or after 45 years) and compared the different clinical characteristics using Chi-square (for dichotomous variables) and Student *t* (for continuous variables, for which we verified that they were normally distributed) tests.

#### 2.3.2 Single SNP association analyses

Genotype and allele frequencies and deviations from Hardy–Weinberg equilibrium (HWE) were estimated with PLINK. ^[21]^ One of these SNPs (rs966423) deviated significantly from HWE in cases (3.2 × 10^−5^), despite not showing an obvious technical genotyping issue (data not shown) and showing >99% concordance between KASP genotypes and available array SNP genotype data in 320 controls (data not shown). This marker was therefore excluded from further analyses. For the remaining 5 SNPs, we calculated odds ratios (ORs) and 95% confidence intervals (CIs) using PLINK and tested both genotypic and allelic models. Association tests were carried out by recruitment center and the combined results were obtained using meta-analyses as previously described. [^22^ ^23^ ^24^] Additionally, we performed analysis between SNP genotypes and the clinicopathological variables as described above.

#### 2.3.3 Multi-SNP analyses

To assess the cumulative genetic risk conferred by these variants, we followed a previously described approach, ^[25]^ in which SNP genotypes are coded as 0, 1, or 2 indicating the number of risk alleles at each locus. The cumulative genetic risk score (CGRS) was calculated using both an unweighted method, which counts the number of risk alleles per individual and a weighted approach (wCGRS), which takes into account the effect size of each risk allele. The average of CGRS and wCGRS between cases and controls were compared with a Student *t* test. To evaluate the classifying power of the logistic regression model with these CGRSs, receiver/operator characteristic (ROC) curves and the area under the curves (AUC) were determined using 2000 stratified bootstrap replicates. All of the statistical analyses were carried out with R.

## 3 Results

### 3.1 Clinical characteristics of Colombian patients with thyroid cancer

Table 1 shows the main clinical characteristics of the 281 Colombian cases who participated in the study. The average age of diagnosis, for PTC, was 46.5 years (standard deviation, SD = 2.37 years) in women and 44.5 years (SD = 5.12 years) in men (*P* = 0.46). For FTC, the average age at diagnosis was 47.6 years (SD = 2.77 years) and 51.3 years (SD = 8.21 years) for women and men (*P* = 0.46), respectively (data not shown). TC was more frequently diagnosed in women (n = 233 cases, 83%) than in men (n = 28, 17%) with a female/male ratio of 4.9:1 in the study. These cases had tumors with a predominant PTC histology (61.2% with PTC and 38.8% with FTC) and about a third of them reported a family history of cancer in first- and second-degree relatives. Most tumors in these cases were small (56.8%), unifocal (63.1%), restricted to one side of the thyroid (80.5%) and did not have capsular invasion (56.4%), vascular invasion (78.4%), or extrathyroid extension (83.4%). Furthermore, most of these tumors lacked lymph node (68.9%) or distal (95%) metastasis. Table 1 also shows comparisons of these TC cases when they were stratified by age at diagnosis (<45 years, n = 111; ≥45 years, n = 170). In these stratified analyses, we found that younger cases were more frequently diagnosed with lymph node metastasis than older patients (47.3% vs. 20.9%, *P* = 2 × 10^−5^, Table 1). We also found borderline significant associations involving histological type, with PTC more common in younger patients and FTC more common in older cases (*P* = 0.082, Table 1) and tumor size, with younger cases presenting more frequently with large tumors when compared to older patients (50.5% vs. 38.5%, *P* = 0.066, Table 1).

**Table 1:**
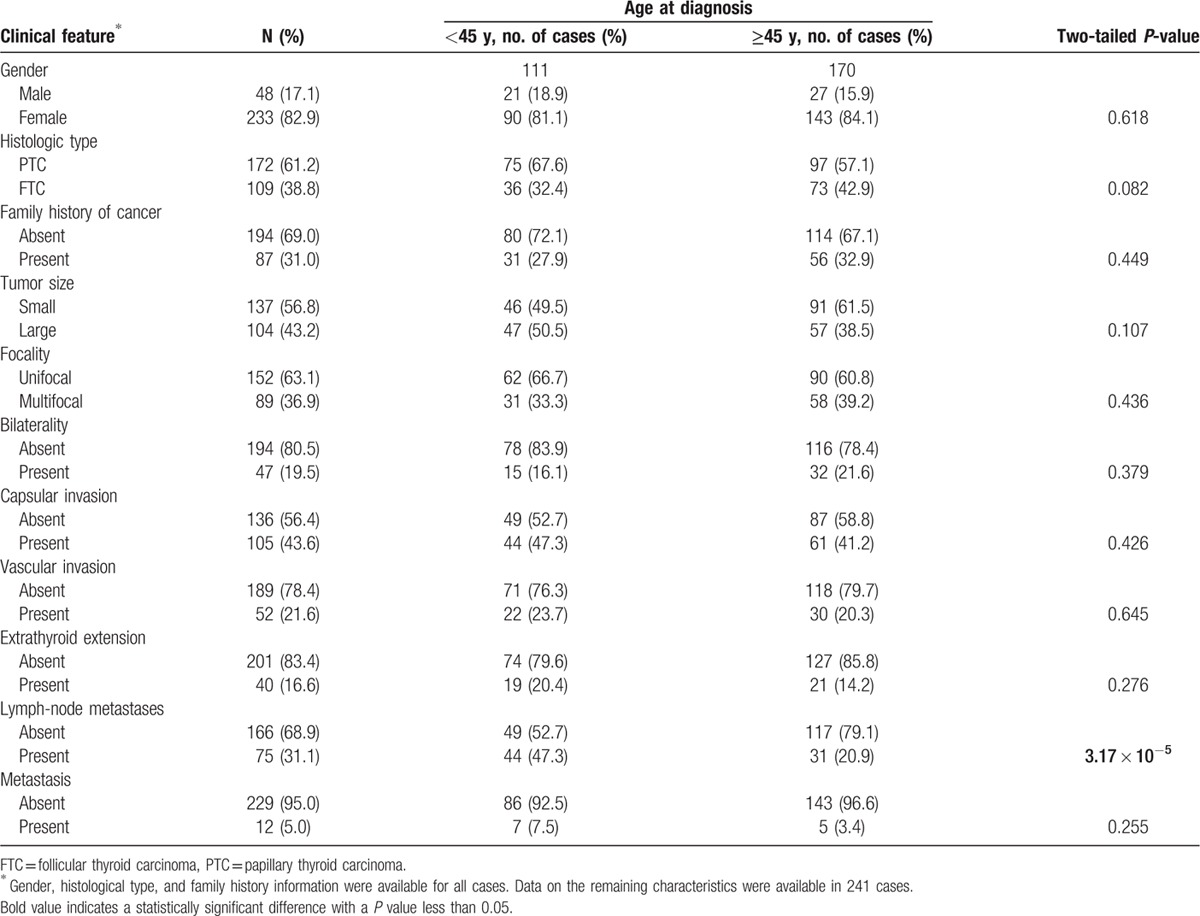
Clinicopathological characteristics of the thyroid cancer cases, stratified by age of diagnosis, included in our study.

### 3.2 Individual associations between known thyroid cancer variants and disease risk

We investigated the individual effects of 5 known TC SNPs on disease risk in our case–control study. The genotype frequencies for these SNPs are shown in Table 2. We detected significant associations between TC risk and rs965513A (OR = 1.41; 95% CI: 1.17–1.71; *P* = 3.0 × 10^−4^), rs944289T (OR = 1.26; 95% CI: 1.05–1.53; *P* = 0.009), rs116909374A (OR = 1.96; 95% CI: 1.09–3.51; *P* = 0.011), rs2439302G (OR = 1.19; 95% CI: 0.99–1.43; *P* = 0.038), and rs6983267G (OR = 1.18; 95% CI: 0.98–1.42; *P* = 0.043). These associations were consistent with previous reports as they involved the same risk alleles identified by European-based studies.[ ^10^ ^11^ ^12^ ^14^ ^15^ ^25^ ^26^ ^27^ ^28^ ^29^ ^30^ ^31^ ^32^ ^33^ ^34^] Therefore, our results indicate that these variants also represent TC risk factors in Hispanics. However, on average these ORs were 10% lower than those reported in Europeans (1.46 vs. 1.60, data not shown), which could be explained by population-specific risk factors and/or by the admixed American Indian and European ancestry of our Hispanic groups.[ ^4^ ^5^]

**Table 2:**
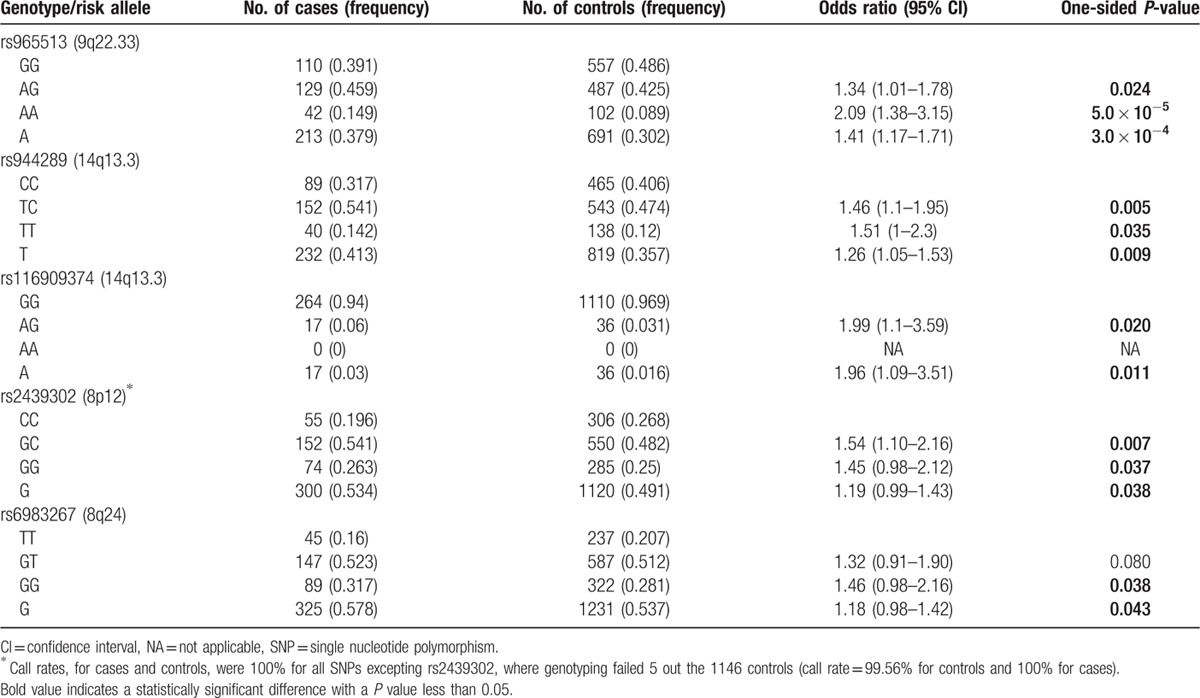
Association statistics between known genetic variants and the risk of thyroid cancer in out Hispanic population study.

### 3.3 Multiallelic associations with disease risk

**Figure 1:**
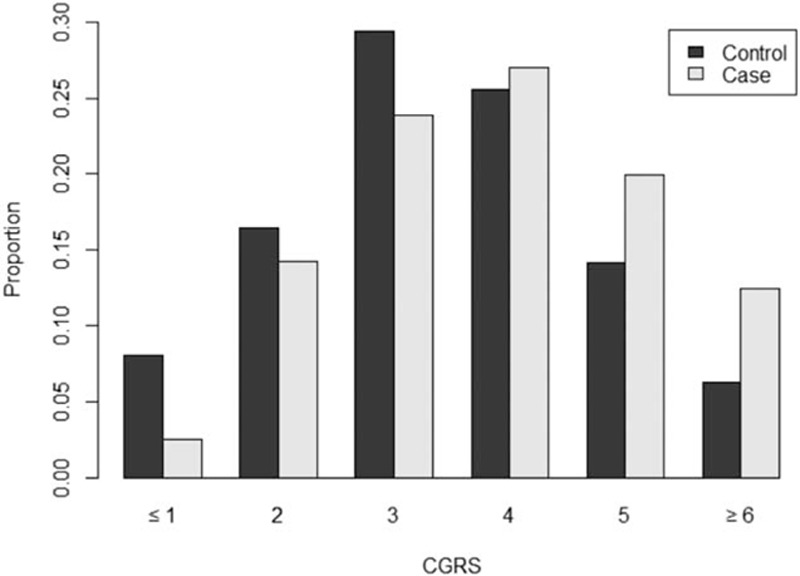
Distribution of number of TC risk alleles in cases and controls included in our study.

In addition to individual SNP analyses, we also estimated the risk of the disease associated with carrying multiple genetic variants. Figure 1 shows the frequency of risk alleles in Colombian TC cases and controls. Consistent with the individual associations, the mean number of risk alleles was higher in cases than in controls (5.16 vs. 4.78, *P* = 4.8 × 10^−6^, Fig. 1). Table 3 shows the risk associated with carrying different number of risk alleles at these loci. We found that there is a 6.33-fold difference in TC risk between individuals carrying 1 or less risk alleles (representing ∼8% of the population) and those with 6 or more alleles (which represented ∼6% of our Hispanic population, Table 3) The association statistics using the weighted approach were similar and suggested that higher polygenic scores were associated with a higher risk of TC (data not shown). These 5 variants, however, did not have enough discriminatory power in the ROC analyses (AUC = 0.60, data not shown).

**Table 3:**
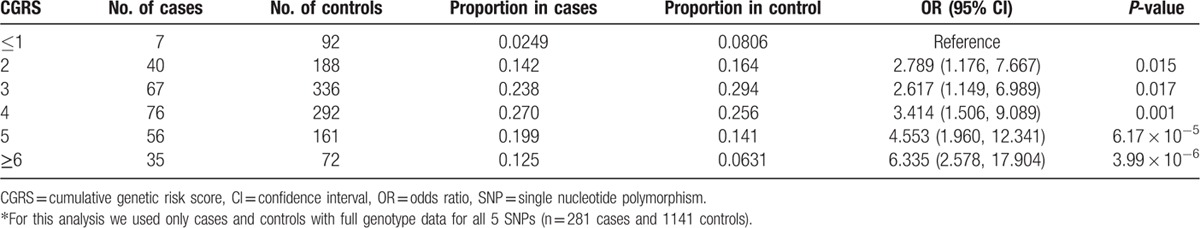
Associations between the cumulative genetic risk score (number of risk alleles) and thyroid cancer in our Hispanic population.

### 3.4 Associations between genetic variants and clinicopathological manifestations

We next explored associations between clinicopathological variables and genetic risk variants. Table 4 summarizes the associations showing nominally significant cases-only *P* values in our study. We found stronger associations between rs944289 and rs116909374 and FTC histology (cases-only *P* values < 0.026 for both SNPs, Table 4). We also found that rs2439302G was more strongly associated in cases with large tumors (cases-only *P* = 0.038) and that rs965513A was more commonly detected in patients with lymph node metastasis (cases-only *P* = 0.018). Although these associations are not corrected for multiple testing, we found that both case–control and case-only analyses were consistent (see Table 4) and suggest that some of these variants are associated with more severe disease. We also tested associations between these genetic risk variants and age of onset (<45 years vs. ≥45 years) using case–control and cases-only models but failed to detect any significant differences in the strength of these associations between groups (data not shown).

**Table 4:**
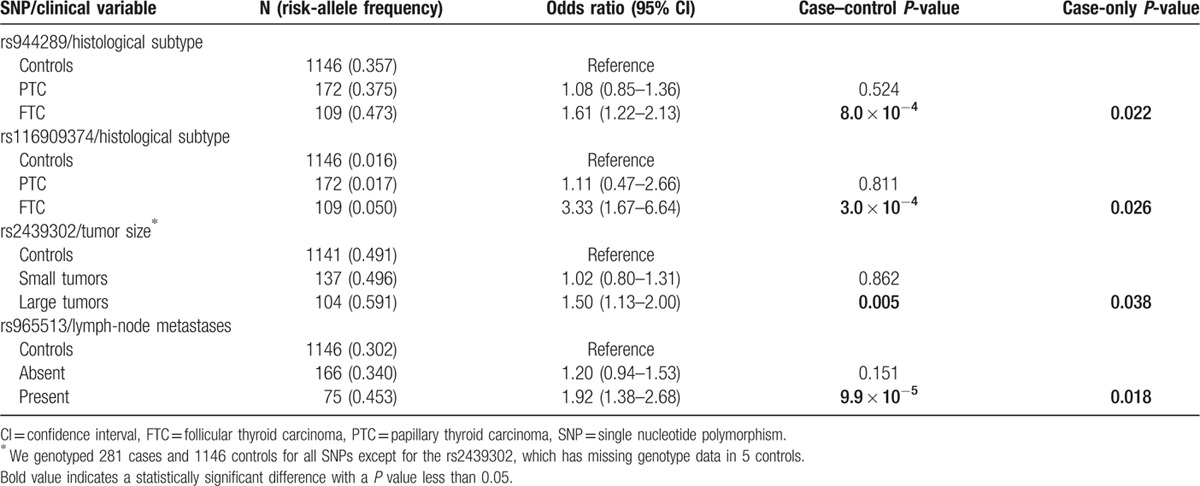
Nominally significant associations between clinicopathological characteristics of our thyroid cancer cases and the risk alleles examined in the study.

## 4 Discussion

Hispanics are the largest population group in the Americas and the largest and youngest U.S. minority. In the United States, after breast cancer, TC is now the second most commonly diagnosed malignancy in Hispanic women; representing 9% of all female cancer cases diagnosed in this minority. ^[3]^ In Colombia, TC is the fifth most common cancer in women from the country. ^[1]^ There is an urgent need to better understand the etiology of this malignancy as it is increasing at an alarming rate in Hispanics. However, TC genetic studies have been carried out so far in populations of European and Asian ancestry and limited information is available on the patterns of TC genetic susceptibility in Hispanic populations. ^[29]^ The aim of the present study was to describe the main clinical characteristics of a cohort of Hispanic TC cases and to evaluate the role of known TC variants in a Hispanic population. To our knowledge, this is the first study where both clinicopathological characteristics and genetic factors are assessed in Hispanics with TC. Our study is important as it contributes toward a better understanding of disease presentation and genetic risk factors in this growing population.

TC is commonly diagnosed in Hispanics from both the United States and Colombia.[ ^1^ ^3^] When comparing the clinicopathological characteristics described in our study to those recently reported for U.S. Whites and Hispanics from California, ^[35]^ we found some commonalities (details shown in Supplementary Table 1). For example, both populations, Hispanics from the United States and from Colombia, have a lower age at TC diagnosis (44 years in U.S. Hispanics and 46.9 years in Colombia) when compared to U.S. Whites (49.7 years ^[35]^). Hispanics also have a lower proportion of male cases (17.7% in U.S. Hispanics, 17.1% in Colombians as compared to 26.3% in U.S. Whites) and a higher mean tumor size (20 mm in U.S. Hispanics, 27 mm in Colombians, and 15 mm in U.S. Whites, Supplementary Table 1). Conversely, we noted some differences between cases from our study and reports in U.S. Hispanics. For example, the higher fraction of older cases found in our study (60.5% cases diagnosed ≥45 years) is more similar to that in U.S. Whites (58.7%) than in U.S. Hispanics (43.3%). Colombian cases also have a rate of regional disease that is nearly 2-fold higher than that reported in U.S. Hispanics (31.15% vs.16.7%, Supplementary Table 1). Hispanics from both Latin America and the United States have a common demographic history that involves a genetic ancestry of American Indians, Europeans, and Africans. ^[5]^ However, we and others have shown that these ancestral proportions vary extensively across Hispanic populations. While Colombians tend to have a slightly higher fraction of African ancestry and similar contributions from Amerindians and Europeans, U.S. Hispanics from California have a predominant Mexican ancestry and hence will have a higher fraction of American Indian ancestry.[ ^4^ ^5^ ^29^] Hispanics living in South America are also likely to have lifestyles and exposures that may differ from those living in North America. While comparing results from our study and published data in the United States are likely to be affected by a myriad of unaccounted sociodemographic factors, the shared ancestry between Hispanics from Colombia and the United States would support the notion that some of these elements may be related to genetic and nongenetic population-specific factors.

The genetic association testing component of our study showed that all 5 known TC SNPs increase the risk of the disease in Colombia. We found that the effect sizes conferred by these variants were consistent with previous reports and hence the risk alleles reported in Europeans and Asians were the same ones increasing TC risk in Hispanics. However, we noted that the effect sizes for these variants were slightly lower in Colombia, which could be explained by differences in exposures, lifestyle, or genetic factors (see above). We showed in the multiallelic analyses that these 5 risk alleles can stratify populations at low and high risk of TC. Having found a >6-fold difference in risk between Hispanics carrying 1 or less risk alleles and those carrying 6 or more risk alleles suggests that these variants will be useful for patient stratification in the future; although it is also clear that additional genetic markers should be identified to increase the discriminatory power of these variants. Our study is important as it validates these SNPs as risk factors in Hispanics. It also suggests that additional Hispanic-based TC genetics studies should be carried out in order to identify additional risk variants in this population, in particular those that may have population-specific American Indian origin. ^[30]^

We found some very interesting associations between these risk variants and indicators of TC severity. Two of these alleles (rs944289 and rs116909374, Table 4) were more strongly associated with FTC, a histological TC subtype that tends to have worse outcomes than the most common PTC form. ^[35]^ We also found that rs2439302 (and rs6983247, as we reported previously ^[3]^), were almost exclusively associated with larger tumors. In addition, rs965513 conferred nearly a 2-fold higher risk of presenting with regional disease. The latter finding is consistent with a recent study that found a stronger association between rs965513 and advanced TC stage. ^[27]^ While these associations were obtained from a relatively small number of cases, they were consistent in both the case–control and the cases-only analyses (see Table 4). We believe that these findings are worthy of follow up in future studies as the identification of TC severity biomarkers are urgently needed. We were surprised to find that lymph node metastasis, a known prognostic factor in older cases, ^[36]^ was more common in younger patients in our study. Interestingly, a recent study showed that lymph node metastasis had adverse impact on overall survival in young PTC patients, suggesting that lymph node metastasis was a prognostic factor for patients of all ages. ^[37]^

In summary, our study characterized the main clinical manifestation of a cohort of Hispanic TC cases. Some characteristics, such as early disease onset and larger female/male ratio, are similar to reports in closely related U.S. Hispanic population and suggest that future studies, comparing migrant and nonmigrant Hispanic groups should be carried out to understand these unique TC manifestations. Our study is also the first that demonstrates that known TC SNPs are risk factors for this common endocrine malignancy in South American Hispanics. Furthermore, we showed that some of these risk alleles have effects on tumor characteristics and prognostic factors. Future studies should attempt to validate our discovery of associations between genetic variants and indicators of disease severity such as FTC histology, tumor size, and presence of lymph node metastasis in other Hispanics and non-Hispanic populations.

## Supplementary Material

Supplemental Digital Content
