# Supplementary material for: Clinical features of Hispanic thyroid cancer cases and the role of known genetic variants on disease risk
Source: Medicine (Baltimore). 2016 Aug 12;95(32):e4148. doi: 10.1097/MD.0000000000004148 (PMC4985291; doi:10.1097/MD.0000000000004148)
Supplement: Supplemental Digital Content [file medi-95-e4148-s001.doc]

Supplementary Table 1. Comparison of clinical characteristic in thyroid cancer cases from Colombia (this study) and similar information previously reported by Harari *et al* (41) with Whites and Hispanics from the United States.

|  | U.S. Whites | U.S. Hispanics | Colombia |
| --- | --- | --- | --- |
| Mean age at diagnosis (y) | 49.7 | 44.0 | 46.9 |
| % > 45y | 58.7 | 43.4 | 60.5 |
| % male | 26.3 | 17.7 | 17.1 |
| Median tumor size (mm) | 15 | 20 | 27 |
| % localized disease | 69.5 | 57.9 | 58.5 |
| % regional disease | 26.3 | 16.7 | 31.1 |
| % metastatic disease | 4.2 | 7.3 | 5.0 |
